# Regrowing the Adult Brain: NF-κB Controls Functional Circuit Formation and Tissue Homeostasis in the Dentate Gyrus

**DOI:** 10.1371/journal.pone.0030838

**Published:** 2012-02-01

**Authors:** Yvonne Imielski, Jens C. Schwamborn, Patrick Lüningschrör, Peter Heimann, Magdalena Holzberg, Hendrikje Werner, Oliver Leske, Andreas W. Püschel, Sylvie Memet, Rolf Heumann, Alain Israel, Christian Kaltschmidt, Barbara Kaltschmidt

**Affiliations:** 1 Molecular Neurobiology, Faculty of Biology, University of Bielefeld, Bielefeld, Germany; 2 AG Stammzellbiologie und Regeneration, Institut für Zellbiologie, ZMBE, Münster, Germany; Bielefeld, Germany; 3 Cell Biology, Faculty of Biology, University of Bielefeld, Bielefeld, Germany; 4 Institut für Molekulare Zellbiologie, Westfälische Wilhelms-Universität Münster, Münster, Germany; 5 Institute of Medical Biology, Immunos, Singapore; 6 Molekulare Neurobiochemie, Ruhr-Universität Bochum, Bochum, Germany; 7 Institut Pasteur, Unité de Mycologie Moléculaire, CNRS URA3012, Paris, France; 8 Institut Pasteur, Unité de Signalisation Moléculaire et Activation Cellulaire, CNRS URA 2582, Paris, France; University of South Florida, United States of America

## Abstract

Cognitive decline during aging is correlated with a continuous loss of cells within the brain and especially within the hippocampus, which could be regenerated by adult neurogenesis. Here we show that genetic ablation of NF-κB resulted in severe defects in the neurogenic region (dentate gyrus) of the hippocampus. Despite increased stem cell proliferation, axogenesis, synaptogenesis and neuroprotection were hampered, leading to disruption of the mossy fiber pathway and to atrophy of the dentate gyrus during aging. Here, NF-κB controls the transcription of FOXO1 and PKA, regulating axogenesis. Structural defects culminated in behavioral impairments in pattern separation. Re-activation of NF-κB resulted in integration of newborn neurons, finally to regeneration of the dentate gyrus, accompanied by a complete recovery of structural and behavioral defects. These data identify NF-κB as a crucial regulator of dentate gyrus tissue homeostasis suggesting NF-κB to be a therapeutic target for treating cognitive and mood disorders.

## Introduction

The hippocampus is crucial for the formation of spatial or episodic memory [1]. In a traditional view the hippocampus was seen as a static structure which is only modified during development. Recently, a new picture of hippocampal plasticity is emerging. Gross anatomical structural changes are induced not only during development but also in adulthood by physiological processes such as learning and pathological processes such as neurodegeneration. Pre-existing neuronal networks undergo modification in connectivity, that include changes in dendritic arborisation and more subtle changes in synaptic density. Completely novel non pre-existing circuits are formed by the addition of adult newborn neurons due to neurogenesis. Thus, neurogenesis is a key-feature of the adult dentate gyrus, but the function of newborn neurons remains unclear [2]. Recent studies suggest that the dentate gyrus (DG) subregion is involved in spatial pattern separation, a process of transforming similar representations or memories into highly dissimilar non-overlapping representations [3], [4].

Neurogenesis is continuing in two brain regions of adult rodent brain, the subventricular zone of the lateral ventricles and the subgranular zone of the hippocampal DG [5], [6], [7], [8], [9], [10]. While many studies have identified molecules and conditions that regulate neurogenesis, the transcriptional control of hippocampal neurogenesis during adulthood is less clear, see [11]. Here, we investigated the role of NF-κB in new circuit formation and structural plasticity.

NF-κB is an inducible transcription factor important for immune function, cancer and anti-apoptotic cell protection [12]. NF-κB is the generic name of dimeric combinations between 5 DNA-binding subunits: p50, p52, c-Rel, RelB, and p65 (RelA), the latter three having transactivation domains. NF-κB comes in two flavours: a cytoplasmic non-DNA binding inducible form, which is a trimeric complex composed of two DNA-binding subunits and one inhibitory subunit of the IkB family; an activated NF-κB is a dimeric complex without the inhibitory subunit, it is transported into the nucleus, where it binds and activates the transcription of target genes. Activation takes place via a kinase cascade, which ends in the IkB kinase complex (IKK), which are specific kinases for the inhibitory IκB subunits. Ubiquitination of IκB leads to its degradation by the proteasome, thus allowing nuclear translocation of NF-κB dimers and activation of their target genes. Activation of NF-κB is signal-driven e.g. by pro-inflammatory cytokines or pathogens.

Within the nervous system, NF-κB is involved in neuroprotection/degeneration, synaptic plasticity [13], [14] in neurite growth [15] and formation of functional dendritic spines [16]. Recently, under pathological conditions such as acute immobilization stress NF-κB could impair neurogenesis after IL-1β-mediated activation [17]. Whereas a lot of information on the role of NF-κB in neuronal process growth is already available, one of the main questions that remained to be answered is: what is the physiological importance of the regulation of axonal growth by NF-κB signaling for neuronal development in vivo [18]. This question will be addressed here in a special brain region, the dentate gyrus.

Previously, a genetic ablation of NF-κB identified a neuronal target gene [19], the catalytic subunit of protein kinase A (PKAc, Prkaca). Taken together some aspects of NF-κB dependent signaling are well established [13], but its function in structural plasticity - that is reorganization of neural circuits [20] by neurogenesis - still remains unclear.

Tissue homeostasis can be defined as the balance between cell death and neurogenesis [21], where stem cells can remodel relevant tissues in response to physiological change [22]. Here we provide evidence in support of the notion that NF-κB regulates tissue homeostasis within the adult dentate gyrus. In the mouse model used here, NF-κB fulfils a dual function: in neuronal progenitors NF-κB is necessary for axogenesis and maturation, whereas in mature granule cells NF-κB regulates neuroprotection as well as synaptic transmission. Thus, inhibition of NF-κB in both developmental stages leads to severe atrophy of the dentate gyrus in aging animals. These data might explain why the neuronal circuit is damaged upon inactivation of NF-κB, because mossy fibers, the axons of granule cells, were degenerating and thus the dentate gyrus can not be regenerated by the addition of newborn neurons.

In this study the most fascinating finding is that re-activation of NF-κB alone, can restore a functional dentate gyrus within the adult brain. Taken together our findings suggest that NF-κB might have therapeutic potential for reversing dentate gyrus dysfunction as observed in Alzheimer's disease and mood disorders.

## Results

### NF-κB controls circuit formation of the mossy fiber pathway and replenishment of the dentate gyrus

**Figure 1:**
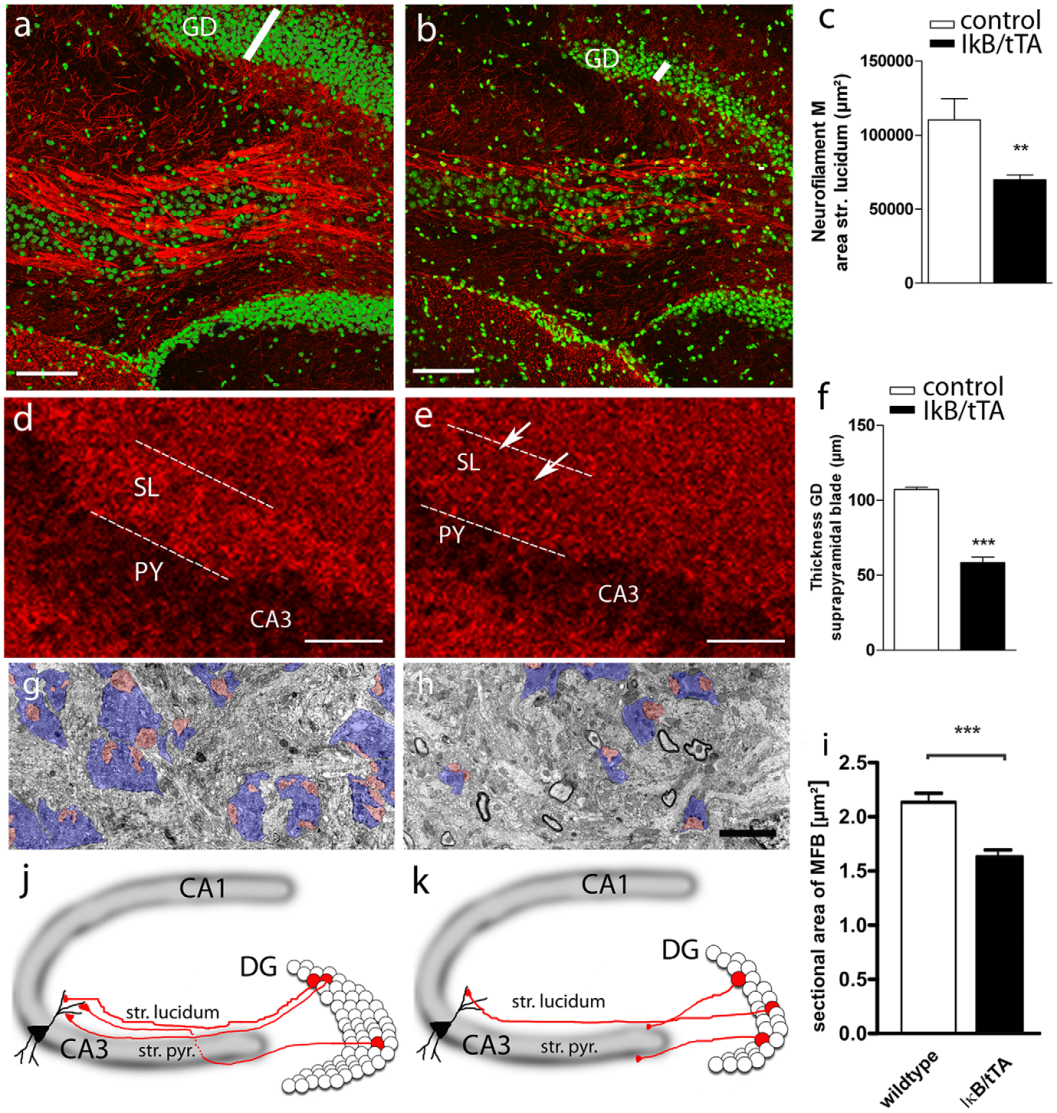
NF-κB regulates mossy fiber projections to CA3 and replenishment of the dentate gyrus. (**a**) Immunostaining for neurofilament M (NF-M) in control mice (IκB/-) reveals a fasciculated organisation of mossy fibers, connecting granule cells to their target cells in CA3. (**b**) Impairment of mossy fiber projections after neuronal NF-κB ablation (IκB/tTA). (**c**) Statistical analysis of NF-M staining shows significantly reduced mossy fiber bundles in IκB/tTA mice (*n* = 8) as compared to controls (*n* = 4) (*P* = 0.0034); (**d**) Synapses stained for synaptophysin are organised in a laminated fashion. Strongest staining was visible in the stratum lucidum (SL), which corresponds to the termination field of mossy fibers on dendrites of CA3 neurons. (**e**) In contrast, staining is strongly reduced in the SL after NF-κB ablation, suggesting an important role of NF-κB in regulating synaptic density. (**f**) NF-κB ablation results also in a significantly decreased dentate gyrus size (white bar in **a**, **b**, *n* = 9) compared to control mice (*n* = 6) (*P* = <0.0001). (**g**, **h**) Electron microscopy shows that size and frequency of mossy fiber boutons (MFB) are reduced after NF-κB inhibition (blue, mossy fiber boutons; red, dendritic excrescences). (**i**) The size of MFB is reduced in IκB/tTA mice. (**j**, **k**) Scheme of structural defects after NF-κB ablation. Scale bars (a,b) 100 µm, (d,e) 20 µm, (g,h) 2 µm); DG-dentate gyrus, SL-stratum lucidum, PY-pyramidal layer, MFB-mossy fiber boutons. Error bars, SEM. ^**^ *P*<0.01; ^***^ *P*<0.0001; Unpaired t-test, two tailed.

In the mouse model used here, neuronal NF-κB ablation can be established by CamKinaseII promoter-driven tetracycline controlled transactivator (tTA) regulating the expression of Super-Repressor IκB in mice (S1). A Super Repressor (SR) of NF-κB was generated by mutating phosphorylation sites in IκB-α. Transgene expression started in transient-amplifying neuronal progenitors (S2) that co-express the early neuronal marker doublecortin (DCX), so called type 2b cells. NF-κB-dependent defects in structural plasticity were severe. Reduction was evident for granule cell axons, the mossy fibers (MF) connecting granule cells with pyramidal neurons of the CA3 region (Fig. 1a,b,c). The thickness of the dentate gyrus (DG) granular layer was reduced by about 50% (Fig. 1f). Pre-synaptic sites of MF were decreased in size and number (Fig. 1 d,e), suggesting an important role for NF-κB in regulating synapse density. Furthermore, we analyzed the morphology of mossy fiber synapses by electron microscopy. After NF-κB ablation, size (by 24%) and number of mossy fiber boutons (by 38%) were reduced, as well as the number of synaptic contacts (by 31%; Fig. 1 g,h,i and S3). Structural NF-κB dependent changes are summarized in Fig. 1 j,k. Thickness of the dentate gyrus was reduced by 50% in 6 months old mice (Fig. 1 a,b) and the mossy fiber projection was impaired, resulting in a reduced synapse density in the stratum lucidum.

We hypothesize that the observed structural changes detected here might be generated by a misbalance of cell death and neurogenesis.

### NF-κB deficient mice show increased apoptosis, neurogenesis and inflammation in the adult dentate gyrus

**Figure 2:**
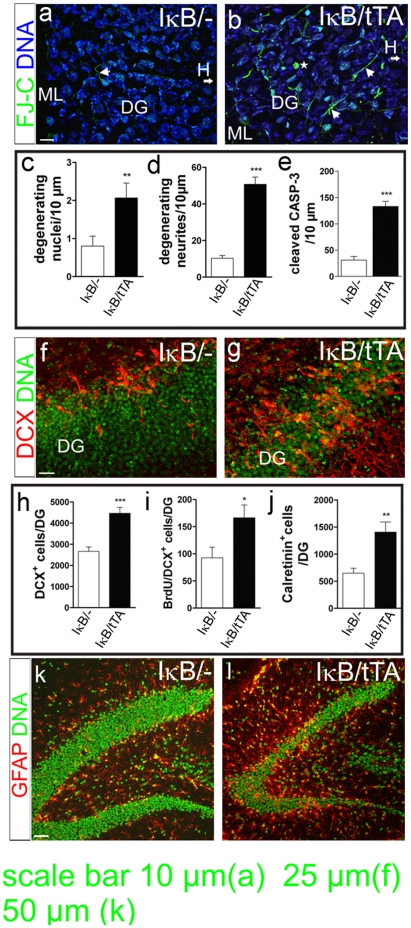
Increased apoptosis, neurogenesis and inflammation in the dentate gyrus of NF-κB deficient mice. (**a**, **b**) Neuronal cell death visualized by Fluoro Jade-C. Degenerating neurites (arrows) and nuclei (asterisk) are significantly increased in IκB/tTA mice (*n* = 3) as compared to control mice (*n* = 3) (**c**, **d**). (**e**) Increased cleaved caspase-3 positive cells after NF-κB ablation (*n* = 3). (**f**, **g**) DCX immunoreactivity is significantly increased in the DG after NF-κB ablation. Note that DCX^+^ cells are not arranged in the subgranular zone alone but migrate deeper into the DG. (**h**, **j**) Total number of DCX^+^ and Calretinin^+^ cells is increased in IκB/tTA mice (*n* = 3) compared to controls (*n* = 3). (**i**) Increased BrdU/DCX^+^ cells in the DG seven days after the last BrdU injections (*n* = 3). (**k**, **l**) Representative images of the distribution of glial fibrillary acidic protein (GFAP) in the hippocampus. Note significantly increased numbers of GFAP positive cells in IκB/tTA mice, particularly within the DG. Scale bars (a, b) 10 µm, (f, g) 25 µm, (k,l) 50 µm; ^*^ *P*<0.05; ^**^ *P*<0.01; ^***^ *P*<0.0001; Error bars: SEM; DG dentate gyrus, GFAP glial fibrillary acidic protein, ML molecular layer, H hilus, DCX doublecortin, FJ-C fluoro Jade-C, BrdU bromodeoxyuridine.

Degenerating neurons in the dentate gyrus were analysed by Fluoro-Jade staining (Fig. 2a,b). NF-κB ablation resulted in an increase of degenerating neuronal nuclei (about 50%, *P* = 0.0093) and neurites (about 70%, *P* = <0.0001, Fig. 2c,d), arguing for a defect in axonal outgrowth. The apoptotic marker cleaved Caspase-3 was increased about 3-fold (Fig. 2e, *P* = 0.0003). We conclude that the net granule cell number is reduced by apoptosis in IκB/tTA mice. Based on previous findings showing that increased mitotic activity is observed after hippocampal damage and healthy mature neurons can inhibit neurogenesis [9], [23] we next investigated changes in neurogenesis.

In controls, newborn neurons are located mainly in the subgranular zone (Fig. 2f), whereas IκB/tTA mice contained misplaced neuronal progenitors (Fig. 2g). NF-κB ablation led to about 30% increase of BrdU^+^ cells (*P* = 0.0233), which were positive for DCX (Fig. 2i) as well. DCX (*P* = <0.0001) and calretinin positive cells (*P* = 0.0019) were increased in IκB/tTA mice (Fig. 2 h,j). Staining for Glial Fibrillary Acidic Protein (GFAP) in NF-κB ablated mice, revealed strongly increased numbers of activated astrocytes (Fig. 2 k,l), a hallmark of neuroinflammation. Within the neurogenic region of the dentate gyrus GFAP positive type 1 neural stem cells are localized in the subgranular zone. Here, GFAP positive cells were detected in an evenly spaced pattern near the dentate gyrus formation and showed a star-like morphology, suggesting that these cells are activated astrocytes.

The observed defects in mossy fiber projection can be explained by two hypotheses: Firstly, apoptosis of already connected mature granule cells or secondly, a defect in axonal specification in newborn neurons.

### NF-κB regulates PKA and FOXO1 expression

**Figure 3:**
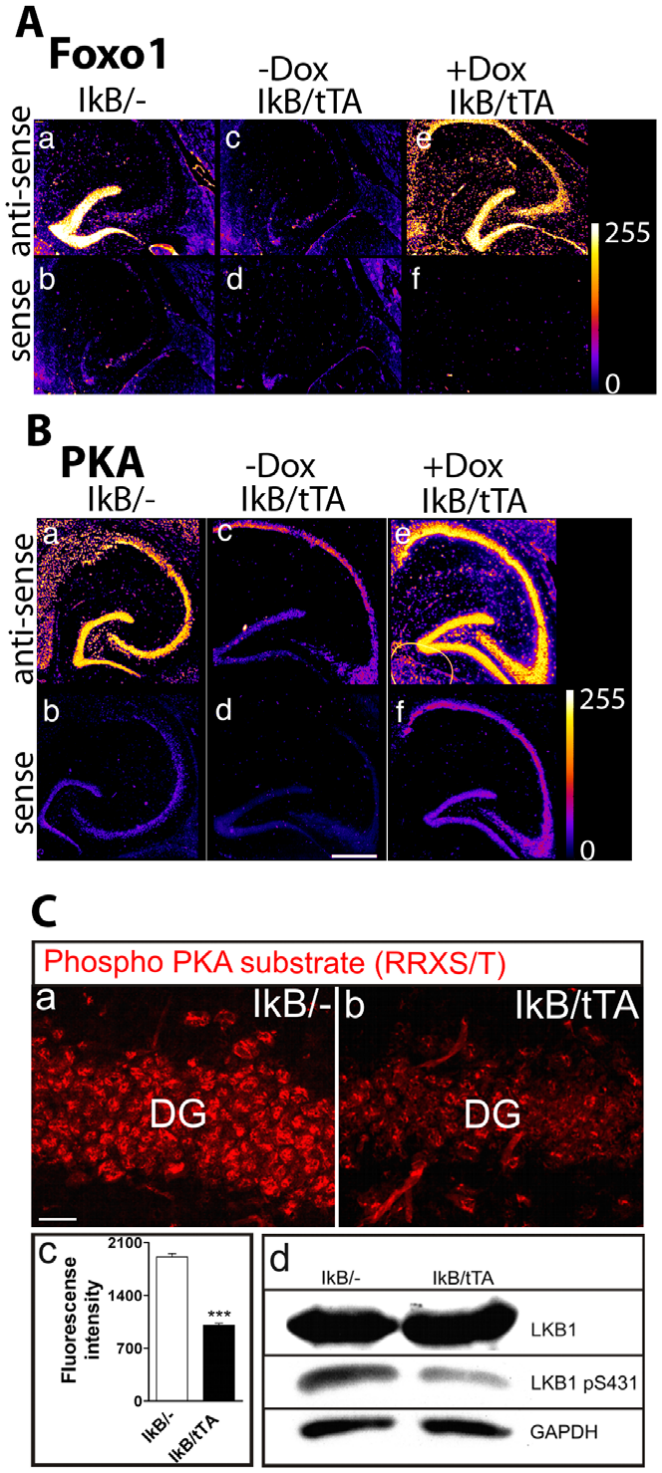
NF-κB dependent regulation of PKA and FOXO1. **A** (**a**, **b**) Forkhead box protein O1 (FOXO1) is expressed in the DG in control mice (IkB/-), but is downregulated in NF-κB deficient mice (**c**, **d**). (**e**, **f**) Doxycycline induced reactivation of NF-κB leads to re-expression of the transcription factor Foxo1 as shown by in situ hybridisation (encoded in false colour). **B** (**a**, **b**) The catalytic subunit alpha of Protein kinase A (PKA) is expressed in the DG of control mice (IκB/-), but is strongly downregulated in NF-κB deficient mice (**c**, **d**). Reactivation of NF-κB by doxycycline treatment results in re-expression of PKA (**e**, **f**); as shown by in situ hybridisation (encoded in false colour, data from [19]). **C** (**a**, **b**) Phosphorylated PKA substrates are reduced in granule cells of the dentate gyrus as detected by phospho-specific antibody. (**c**) Fluorescence intensity is significantly decreased in IκB/tTA mice (*n* = 4) as compared to controls (*n* = 4) (*P* = <0.0001) (**d**) Phosphorylation of LKB1, a substrate of PKA, is reduced in hippocampal brain extracts from IκB/tTA mice as detected by Western blotting. Scale bar (a, b) 20 µm; Error bars: SEM; DG dentate gyrus.

To identify potential NF-κB target genes involved in these processes, we performed microarray analysis [19] and examined the expression of two relevant NF-κB target genes, Foxo1 and the catalytic subunit of Protein kinase A (PKA) (HUGO gene name: Prkaca). While Foxo1 expression is restricted to the dentate gyrus in controls (Fig. 3A a,b), Foxo1 expression is no more detectable after NF-κB ablation (Fig. 3A c,d). Re-activation of NF-κB by doxycycline can transcriptionally switch on Foxo1 but extends the area of expression to CA3 and CA1 (Fig. 3A e,f). Interestingly, a recent report showed an involvement of Foxo1 in axonal outgrowth in hippocampal cultures and in the developing cerebellum [24]. Furthermore, also PKA expression is regulated by NF-κB (Fig. 3B). PKA expression in controls was detected in all subfields of the hippocampus, whereas NF-κB ablation resulted in a complete loss of PKA in the dentate gyrus and in CA3. Re-activation of NF-κB by doxycycline resulted in expression of PKA similar to controls. Since PKA plays a crucial role in axon fate determination [25] and is a neuronal target gene of NF-κB, we have analyzed PKA activity after NF-κB ablation. Here we show that PKA activity is reduced by about 50% in the DG (*P* = <0.0001) (Fig. 3C a,b,c). In extension, phosphorylation of the PKA substrate LKB1 at Ser 431 was reduced after NF-κB ablation (Fig. 3Cd), correlating with a down-regulation of the catalytic subunit of PKA in this mouse model.

### Cell autonomous role of NF-κB in axogenesis

**Figure 4:**
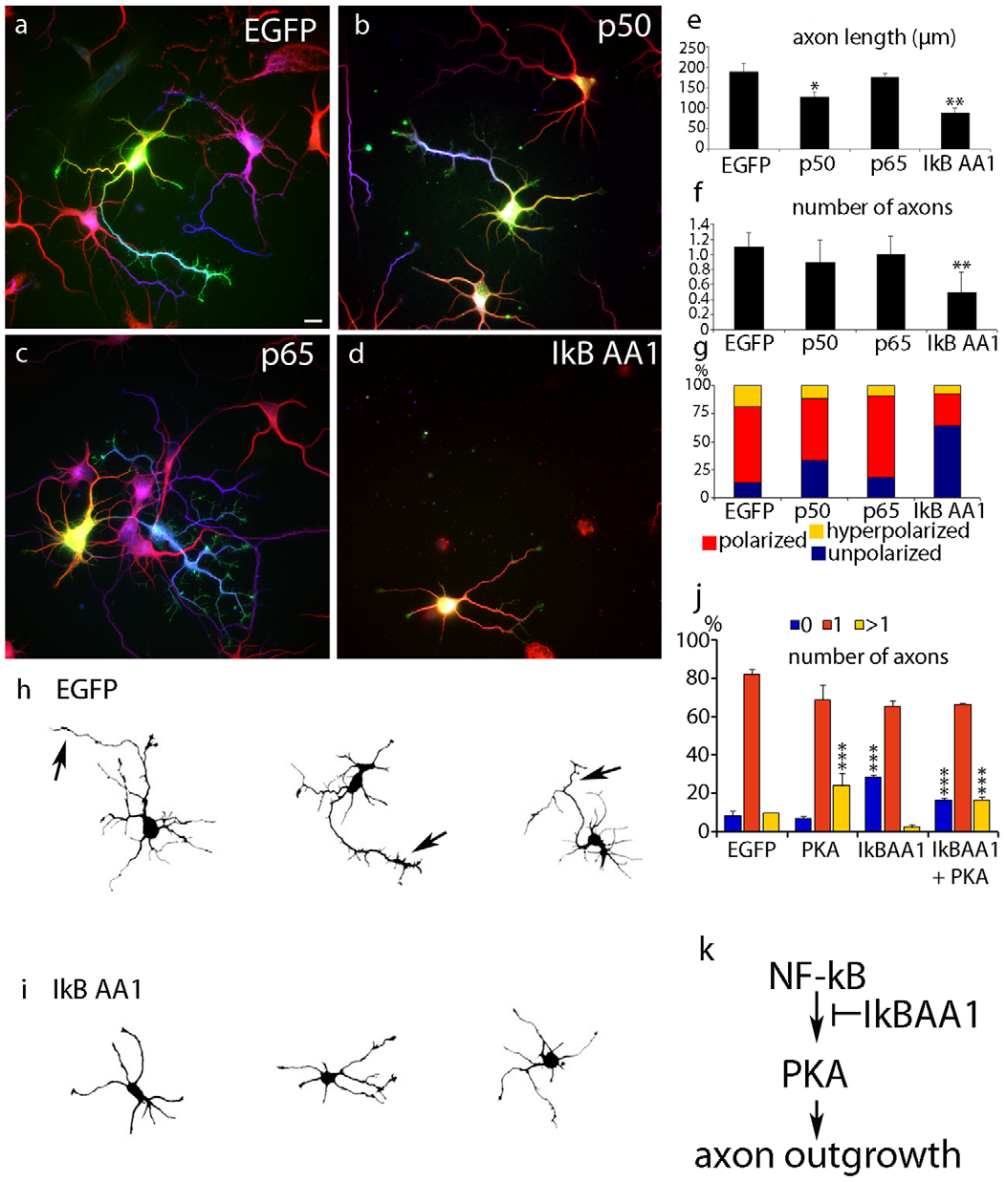
NF-κB dependent changes in axonal fate determination in vitro. Effect of NF-κB inhibition in hippocampal neurons transfected 2 hours after plating with expression vectors for EGFP and NF-κB subunits as indicated. The analysis of NF-κB induced changes in morphology was done by immunostainings with markers for neuronal polarity. Tau-1 immunoreactivity was used as axonal marker, minor neurites (dendrites) were identified by anti-MAP2 labelling. Axon length was reduced in neurons transfected with super-repressor-IκB (from 188 µm±20 µm in the EGFP control to 87 µm±10 µm after IκB transfection (**d**, **e**). To a lesser extend shorter axons was observed in neurons transfected with the p50 subunit (**b**, **e**). Thus super-repressor-IκB expression resulted in neurons without Tau-positive axons (**d**). Control neurons acquired a normal neuronal morphology (**a**, **g**) and extended a single axon (1.1±0.2) plus about 4 minor neurites as shown before (Schwamborn et al., 2007; Schwamborn and Püschel, 2004). (**g**) Transfection with NF-κB DNA-binding subunits p50 and p65 did not induce a significant change in axon number (p50: 0.9 axons per cell±0.3; p65: 1.0 axons per cell±0.2). (**h**, **i**) Representative samples of reconstructed hippocampal neurons. Neurons transfected with control plasmids (**h**) and with super-repressor IκBα-AA (**i**). Note the strongly reduced complexity of neuronal processes in NF-κB ablated cells, resembling an immature developing neuron. (**j**) Furthermore we tested, if the effect on neuronal morphology could be rescued by the NF-κB downstream target gene PKAc. Indeed, overexpression of PKAc in hippocampal neurons showed hyperpolarized neurons with multiple axons, consistent with the recent observation of PKA dependent phosphorylation of the downstream kinase LKB1, which is necessary for axon differentiation. Coexpression of super-repressor-IκB and PKAc led to a hyperpolarized phenotype. Transfected cells were identified via EGFP fluorescence. Development of neuronal polarity was analyzed by determining the length of minor neurites per cells (**e**). Error bars: SEM; *** p<0.0001 compared to EGFP; *n* = 3 independent experiments. (**k**) Model of a transcriptional network regulating axon outgrowth of the granule cells. NF-κB can activate the transcription of the catalytic subunit PKAc alpha, which is necessary for axon formation. NF-κB activation can be inhibited by overexpression of super-repressor IκBα-AA, which interferes with PKA transcription and axon formation. Error bars: SEM; *P*<0.05; ^**^ *P*<0.01; ^***^ *P*<0.0001.

To analyse a potential cell autonomous function of NF-κB, axogenesis in hippocampal cultures was studied. In accordance to the situation in vivo, IκB expression inhibits axogenesis in vitro as well (Fig. 4a–d; h–i). The analysis of NF-κB induced changes in morphology were examined by markers for neuronal polarity. Tau-1 immunoreactivity was used as an axonal marker, minor neurites (dendrites) were identified by anti-MAP2 labelling. Axon length was reduced in neurons transfected with super-repressor-IκB (from 188 µm±20 µm in the EGFP control to 87 µm±10 µm after IκB transfection) (Fig. 4 e). To a lesser extent shorter axons were observed in neurons transfected with the p50 subunit (Fig. 4 b, e). This might reflect the capability of p50 to act as a repressor, lacking its own transactivation domain. Thus super-repressor-IκB expression resulted in neurons without Tau-positive axons (Fig. 4 d,i). Control neurons acquired a normal neuronal morphology (Fig. 4 a, g) and extended a single axon (1.1±0.2) plus about 4 minor neurites. Transfection with NF-κB DNA-binding subunits p50 and p65 did not induce a significant change in axon number (p50: 0.9 axons per cell±0.3; p65: 1.0 axons per cell±0.2), (Fig. 4 g). Camera lucida tracing of neurons transfected with super-repressor-IκB, revealed a clearly visible defect in axon formation (compare Fig. 4 h with 4 i). Taken together these data provide evidence, that NF-κB controls axogenesis in vivo and in vitro. To study a role of the NF-κB target gene PKA, we used again neuronal polarity assays in hippocampal cell cultures. Overexpression of PKAc in hippocampal neurons induced the formation of supernumerary axons (Fig. 4j and S4), consistent with the PKA-dependent phosphorylation of LKB1 [25]. Finally, co-expression of IκB and PKAc led to a phenotype with multiple axons, arguing for a position of PKAc downstream of the NF-κB pathway (Fig. 4 k).

### NF-κB dependent impairment of spatial pattern separation can fully be restored by NF-κB reactivation

**Figure 5:**
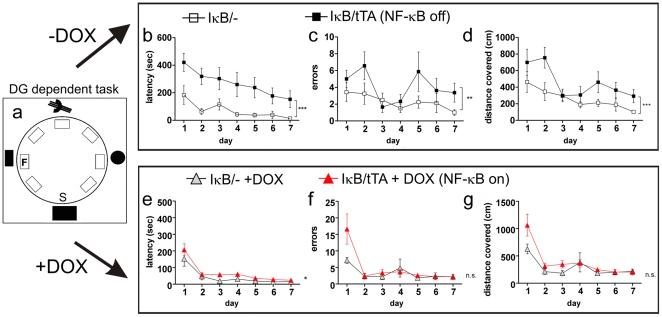
Impaired NF-κB signalling interferes with spatial pattern separation. A *spatial pattern separation-Barnes Maze (SPS-BM)* was developed to test DG dependent spatial pattern separation (**a**). During consecutive days of training the mice had to find the food house (location F), but only one of the identical seven houses on the plate is freely accessible. Dependent on distal extramaze cues the animals have to find the open food house. The presentation of several identical objects in an environment should lead to overlapping activation in place cells. The analysis of the SPS-BM shows highly significant memory deficits in NF-κB ablated mice (*n* = 9) as compared to controls (*n* = 8). Latency (**b**), distance (**d**) and error rate (**c**) were significantly increased in IκB/tTA mice. Reactivation of NF-κB in IκB/tTA animals by doxycyline treatment (*n* = 13; lower panel) improved the performance considerably. Mice showed significantly decreased latency (**e**), the error rate (**f**) and distance covered (**g**) was comparable to control mice (*n* = 6). two-way ANOVA evaluation; error bars: SEM; S = Start position; F = food house.

We hypothesized that the observed NF-κB dependent structural plasticity in the DG affects behavior. In classical *Barnes Maze* (*BM*, [26]), NF-κB dependent behavioral defects were not detectable (S5). Therefore, we developed a novel challenging spatial task, based on the *Barnes Maze*, in which animals have to differentiate between locations with subtle differences (*Spatial-Pattern-Separation-BM: SPS-BM*). On the circular platform of *SPS-BM* mouse houses containing food were placed evenly, but only one house contained an accessible food reward (scheme in Fig. 5). Mice were analyzed in *SPS-BM* for seven days with one trial per day. IκB/tTA mice needed considerably more time to reach the food house F (latency, *F*_1,6_ = 40.94, *P*<0.0001), covered more distance (*F*_1,6_ = 16.13, *P* = 0.0001) and made more errors (*F*_1,6_ = 7.244, *P* = 0.0085) (Fig. 5, upper panel). Strikingly, after re-activation of NF-κB by doxycycline treatment for four weeks, IκB/tTA mice could learn the behavioural task as well as controls (latency, *F*_1,6_ = 5.150, p = 0.0251; errors *F*_1,6_ = 1.784, p = 0.1841; distance *F*_1,6_ = 3.613, p = 0.0598 (Fig. 5 lower panel) (see S7). Taken together these data argue for a functional recovery of the dentate gyrus by re-activation of NF-κB signaling as measured by the SPS-BM. Furthermore we analyzed the cellular basis of this improvement in long-term memory. According to these results, *SPS-BM* seems to be a novel dentate gyrus-dependent behavioral task for spatial pattern separation.

To measure the general learning ability of IκB/tTA mice, we analyzed the mice in a classic BM task with visible external cues. One advantage of the *BM* is the possibility to analyze the animal's search strategies: random, serial and spatial [26]. Control mice learned the task by starting with random-, followed by a serial- and ending with spatial-search strategy (Fig. S7). In contrast, IκB/tTA mice used random and serial search strategies by chance, but never learned a spatial search strategy. Although the IκB/tTA mice with NF-κB ablation were able to learn the classic *BM* and acquired spatial orientation (S6 a–c; latency *F*_1,7_ = 1.585, *P* = 0.2111, distance covered *F*_1,7_ = 0.03702, *P* = 0.8478 and errors made *F*_1,7_ = 0.05903, *P* = 0.8086), similar to controls. Taken together these data show that newborn neurons are required for spatial pattern separation and learning to navigate in a world with subtle environmental differences, processes where NF-κB integrates structural changes with spatial pattern separation.

### Reactivation of NF-κB leads to regrowing of the dentate gyrus and recovery of structural defects

**Figure 6:**
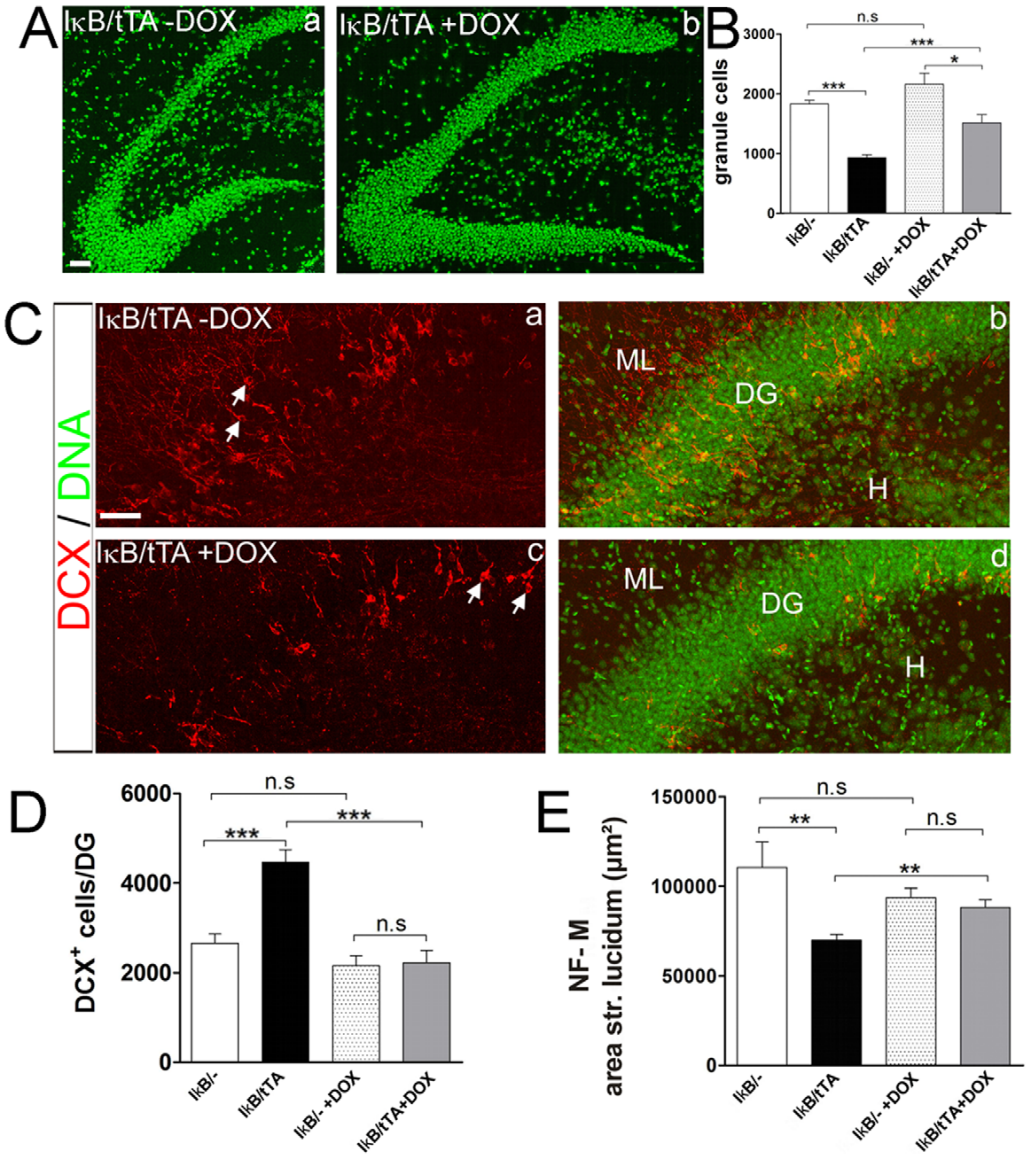
Reactivation of NF-κB leads to regrowing of the dentate gyrus. (**A**) (**a**) The size of the dentate gyrus (DG) in NF-κB ablated mice is strongly reduced. Reactivation of NF-κB leads to increased number of granule cells within the DG (**b**). (**B**) Statistical analysis shows a significant decrease of granule cells after NF-κB ablation (IκB/tTA mice, *n* = 9; as compared to control mice IκB/-; *n* = 3, (*P* = <0.0001)). Note, after reactivation of NF-κB by doxycycline treatment the number of granule cells is increased in IκB/tTA mice (*n* = 7) (*p* = 0.0002). No difference in the control groups IκB/- (*n* = 3) and IκB/- +DOX (*n* = 3) was detected (*p* = 0.0605). (**C**) Doublecortin (DCX) staining marking neuronal precursors. After doxycycline treatment the number of DCX positive cells is strongly decreased in IκB/tTA mice (*n* = 3) (**c**, **d**) as compared to NF-κB ablated group (*n* = 3) (**a**, **b**). (**D**) Statistical evaluation shows significantly less DCX positive cells after doxycycline treatment in IκB/tTA mice (*n* = 3) as compared to untreated IκB/tTA group (*n* = 3) (*p* = <0.0001). No difference in control groups IκB/- (*n* = 3) and IκB/- +DOX (*n* = 3) was detected (*p* = 0.1063). (**E**) Statistical analysis of neurofilament-M (NF-M) staining shows significantly reduced mossy fiber bundles in IκB/tTA (*n* = 8) mice as compared to controls (*n* = 4) (*p* = 0.0034). Reactivation of NF-κB results in mossy fiber outgrowth of newborn neurons, thus increasing the area of mossy fibers in doxycycline treated IκB/tTA mice as compared to the untreated group (*p* = 0.0024, t-test with Welch's correction). No difference in the control groups IκB/- (*n* = 4) and IκB/- +DOX (*n* = 3) was detected ( *p* = 0.1897). Error bars: SEM; *p*<0.05; ^**^ *p*<0.01; ^***^ *p*<0.0001.

Recently several studies suggested, that addition of new cells by neurogenesis improved learning capacity in spatial pattern separation [27]. Therefore we tested in our mouse model, whether the behavioral benefit observed after reactivation of NF-κB might have a cellular origin. Thus we studied the cellular effects of NF-κB reactivation in our mouse system where the expression of IκB can be repeatedly turned on and off non-invasively. After four weeks of treatment with doxycycline, mice from the behavioral analysis were analyzed histologically. NF-κB dependent re-growth of the dentate gyrus was detected by counting the number of nuclei (Fig. 6A,B) from seven animals. These mice, living for a period of 6 months without NF-κB activity in the hippocampus, showed severe dentate gyrus atrophy (Fig. 6B (compare IκB/- with IκB/tTA, p = 0.0001). Reactivation of NF-κB led to a re-expression of the downstream targets Foxo1 and PKA (see Fig. 3 A, B) and a significant (p = 0.0002) increase in cellular number by about 60% (Fig. 6A,B). Surprisingly, NF-κB regulated re-growing of the dentate gyrus was still possible even in an already degenerating dentate gyrus. Doublecortin (DCX) positive cells were generated at large numbers after NF-κB ablation, due to a disequilibrium between apoptosis and neurogenesis (see above). Therefore we analyzed now, whether NF-κB activation could rescue this disequilibrium. We compared the amount of DCX positive cells after reactivation of NF-κB in relation to controls (Fig. 6). In mice with NF-κB ablation, DCX positive cells were dispersed all over the dentate gyrus (Fig. 6C, IκB/tTA, arrows). However, reactivation of NF-κB mediated the appropriate location of those DCX-positive cells within the subgranular zone (see arrows). From these data we conclude that NF-κB directs migration of DCX-positive neuronal progenitors in the dentate gyrus. In addition to the appropriate position of DCX positive cells, their number was now strongly reduced (Fig. 6D) after reactivation of NF-κB (by about 47%, p<0.0001). There was no significant difference compared to DOX treated controls.

Finally, we investigated circuit formation by NF-κB dependent axonal outgrowth of mossy fibers (Fig. 6E). A significant (p = 0.0024) increase of neurofilament positive mossy fibers was detected in mice after NF-κB reactivation (IκB/tTA +DOX). There was no significant difference when compared to DOX treated controls. Taken together the NF-κB dependent rebuilding of the dentate gyrus and its mossy fiber projection suggests a cellular basis for the observed improvements in behavior.

## Discussion

### NF-κB controls circuit formation and tissue homoeostasis

Here we used behavioral analysis, involving a newly developed SPS Barnes maze and morphological analysis to study the role of NF-κB in the hippocampus. We found that NF-κB signaling is crucial for tissue homeostasis and functional circuitry of the dentate gyrus during aging. Immunocytochemistry and electron microscopy of the mossy fiber projections and synaptic contacts with CA3 dendrites point to a role of NF-κB in axogenesis and synaptogenesis in vivo and in vitro. Formation of mossy fiber boutons was significantly reduced in size and number after NF-κB ablation. Neuronal NF-κB might have two functions: In immature neurons NF-κB is necessary for axon formation, survival and integration into the neuronal network, whereas in mature neurons NF-κB is important for survival and synaptic activity [13], [28]. Previously, structural defects and increased cell death after long time NF-κB inhibition were overlooked [29], [30], presumably due to mainly analyzing young animals and lack of appropriate cell death assays. Inhibition of both functions resulted in severe defects of tissue homeostasis during aging in the dentate gyrus. A rescue by increased neurogenesis did not regenerate the dentate gyrus, since NF-κB ablation blocked the differentiation from a type 2b neuronal precursor to a mature neuron, resulting in a net reduction in cell number.

Recently, the role of NF-κB p50 in neurogenesis was analyzed [31], in these p50^−/−^ mice the net rate of neural precursor proliferation was unchanged, but only 50% of newborn neurons survived in the DG and a defect in spatial short term memory was observed. Because in p50^−/−^ mice only one NF-κB subunit is deleted in all cell types, we used in this study a neuronal-specific ablation of all NF-κB subunits. We confirmed in part the results of [31] for a role of NF-κB in neurogenesis and extended that conclusion to a function of NF-κB in tissue homeostasis, axogenesis and spatial pattern separation. In contrast, here we find an increased rate of proliferating DCX-positive granule cell progenitors, presumably due to the high rate of apoptosis observed in DG (see [32] for discussion of feedback mechanisms). These data suggest, that regrowing after re-activation of NF-κB has a major impact on integration and survival of newborn DCX^+^ neurons. The regulation of adult neurogenesis by transcription factors is still a matter of debate [33] and our data show that the transcription factor NF-κB is a crucial regulator of neurogenesis, essential for axogenesis and integration of newborn neurons.

### Molecular mechanism controlling NF-κB directed axogenesis

A role for NF-κB in neurite outgrowth was first described in cultured nodose neurons, furthermore size and complexity of cortical pyramidal dendrites was reduced after NF-κB inhibition [34]. Recently, it was shown that dendritic spines from hippocampal cultures were regulated in density by NF-κB p65 [16]. Similarly a p50 mediated repression of Notch ligand Jagged is responsible for neurite remodeling [35].

**Figure 7:**
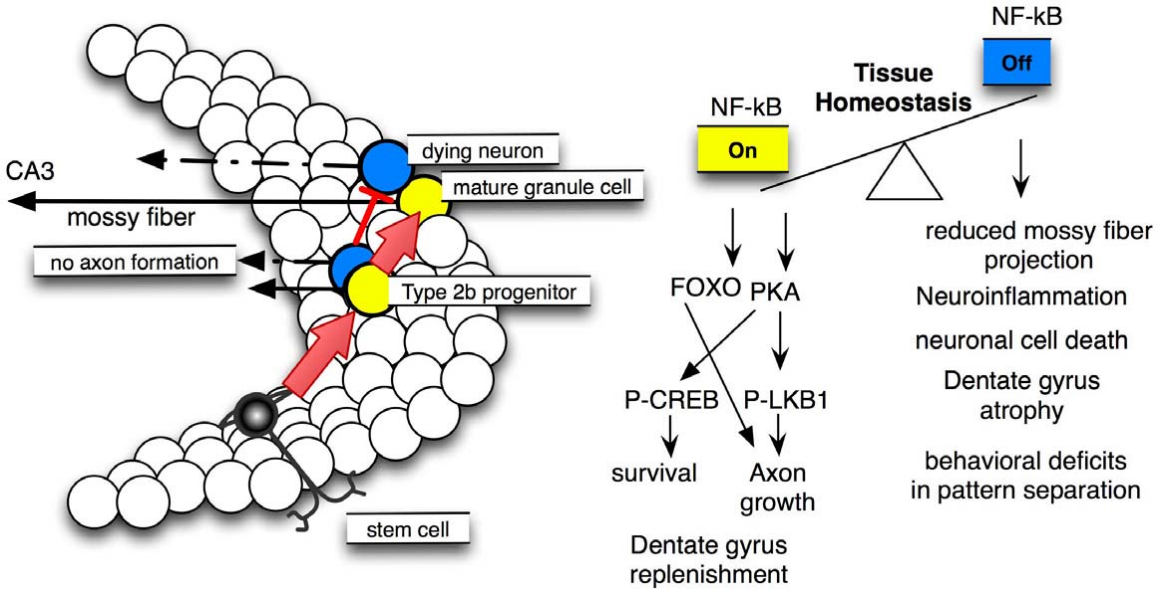
NF-κB signalling can play different roles in dentate gyrus tissue homeostasis. The dentate gyrus (left) is built from stem cells that develop to neuroblasts and type 2b progenitors that still can divide. These progenitors give rise to mature granule cells, connecting CA3 pyramidal neurons via mossy fibers. NF-κB signalling plays an important role at least in two of the processes that regulate the balance between degeneration and regeneration (tissue homeostasis) of the dentate gyrus. That is firstly axon formation and maturation from a immature type 2b neuronal progenitor (DCX^+^) to a mature granule cell neuron and secondly regulation of survival and energy balance of mature granule cells. yellow = cells with NF-κB on; blue = NF-κB ablated cells, NF-κB *off*. Right side, molecular mechanisms: Our results point to a cell autonomous mechanism by which NF-κB regulates axogenesis in newborn granule cells. Critically involved are the FOXO transcription factors and the bona fide NF-κB target gene PKA (Prkaca). A second role for NF-κB in mature granule cells is neuroprotection presumably transmitted by a PKA – phospho-CREB pathway. Ablation of NF-κB (NF-κB off) results in severe structural and behavioural defects described above.

While the function of NF-κB in the postsynaptic compartment is quite well established the role of NF-κB in presynapses and axon outgrowth is still unclear [18]. Here we uncovered a NF-κB dependent signaling pathway relevant for axogenesis (see Fig. 7), involving the forkhead transcription factor Foxo1 and a PKA kinase cascade, which might integrate both: axogenesis and neuronal survival. The NF-κB target gene PKAc phosphorylates CREB [29], which was shown to increase survival of newly generated granule cells [36]. Additionally, LKB1 is a substrate of PKA and is an important player in axogenesis [25], [37]. Phosphorylation of LKB1-S431 by PKA, which is a crucial step for initiation of axon outgrowth, is decreased in NF-κB ablated mice. In this line we show that Foxo1 exclusively is expressed in immature and mature granule cells of the dentate gyrus and its expression is strongly dependent on NF-κB signaling. FOXO1 signaling together with other members of the FOXO family was recently reported to regulate axogenesis, both in cerebellar granule cells and in hippocampal cell cultures and within the cerebellar cortex [24]. In addition recent reports suggest an involvement of FOXO in neural stem cell homeostasis and energy balance [38], [39]. Thus, NF-κB might function as a master regulator upstream of several other transcription factors such as CREB and FOXO. The strong effect of NF-κB on tissue homeostasis of the dentate gyrus might occur only if two circumstances happen together: enhanced cell death coupled with a blockade in neuronal progenitor differentiation, which normally could improve the deficit. That might be the reason why NF-κB dependent tissue homeostasis was observed in the nervous system for the first time. Recently, it was shown that NF-κB regulates tissue homeostasis in several other organs such as skin, liver and gut [40].

Taken together we show that NF-κB plays an important role at three consecutive stages of neurogenesis: proliferation/differentiation of neural progenitor cells, axon specification and integration of young neurons and survival of mature granule cells. A similar conserved function at different stages of neurogenesis has been previously described for members of the TRIM-NHL family [41], [42].

Currently two different models are discussed for the function of new neurons and memory capacity [2] a “replacement model” where a newborn neuron replaces an already existing one and a second “addition model”, where new neurons are added to the existing neuronal network. Our data suggest a function of NF-κB in favor of the “replacement model”. Despite the vast increase in newborn neurons, DG tissue homeostasis is disturbed, resulting in a net reduction of granule cell number by about 50% and finally to an atrophy of DG during adult life. NF-κB ablation was not toxic in vivo [29], [43] and in vitro on its own, but granule cell precursors do not survive in vivo presumably due to a failure in axon formation by reduction of phospho-LKB1.

### NF-κB dependent integration of new born neurons is crucial for spatial pattern separation

The observed NF-κB dependent structural defects resulted in a behavioural phenotype. Recently, behavioural tests [27] were developed to measure memory of subtle differences in spatial environment. The authors suggest that neurogenesis enhanced the recognition of lowly separated cues. In addition [44] have shown that Bax ablation increased neurogenesis and resulted in improved discrimination between similar contexts. In this line, we developed a special behavioral test (*SPS-BM*), which is able to measure spatial pattern separation with the advantage to analyze search strategies. IκB/tTA mice revealed a severe impairment in this *SPS-BM*. They progressed only through the first two steps of learning, using random and serial search strategies by pure chance, but failed to use the spatial strategy consistently. In contrast a spatial search strategy might require the mouse to learn multiple relationships among extramaze cues to guide it to its target, the food house.

On the other hand IκB/tTA mice could learn tasks such as the classic *BM* and the *Morris water maze* (*MWM*) to some extent [19]. This is in accordance with previous results showing that the DG is mainly responsible for spatial pattern separation tasks [2], but is not involved in navigation via the temporoammonic pathway (see S5; [45]).

The mouse model described here is a phenocopy of Alzheimer's disease (AD) in three aspects: reduced NF-κB activity as in AD brains [46] and increased proliferation of immature DCX^+^ neuronal precursors and progressive cell loss [47] coupled with strong neuroinflammation [48]. Thus re-activation of NF-κB might be an interesting therapeutic strategy for neuro-regeneration of the adult dentate gyrus in the future.

## Experimental Procedures

### Animals

Mice were kept under specific pathogen free conditions as defined by the Federation European Laboratory Animal Science Association (FELASA) in the central animal facility of Bielefeld University. Mice were kept in standard cages in a temperature and humidity controlled (22°C) room under diurnal condition (12 h light/dark cycle), with HEPA filtered air. Food and water were provided *ad libitum*. IκB/tTA mice were described in [19], [29], genotyping was done by PCR. To reduce individual variability only males with an age difference of less then 2 weeks were used in behavioural experiments. For NF-κB re-activation experiments, doxycycline (Dox) was administered in drinking water (2 mg/ml w. 2,5% sucrose) for 14 days. All animal experiments were approved by the governmental animal and use comitee, LANUV, Düsseldorf of the state North Rhine-Westphalia under license number 8.87–51.04.20.09.317 (LANUV, NRW).

### Preparation of tissue

For frozen sections animals were decapitated, brains were removed and embedded in TissueTek OCT compound, frozen in −20°C cold 2-methylbutane and cut horizontally on a Leica cryotome. Sections of 12 µm thickness were mounted on Superfrost slides, dried for 5 min at room temperature and stored at −80°C until use.

For fixed sections, animals were anesthetized with Avertin and transcardially perfused with phosphate buffered saline (PBS) containing heparine (0.025 g/100 ml PBS) and procaine (0.5 g/100 ml PBS) followed by 4% paraformaldehyde in PBS. Subsequently brains were postfixed in 4% paraformaldehyde at 4°C for 24 h and dehydrated in 30% sucrose in phosphate buffer saline at 4°C. 40 µm horizontal sections were cut on a Leica Frigocut cryotome and stored in cryoprotectant solution (0.1 M phosphate buffer, 50% glycerol, 0,14% MgCl_2_, 8.6% sucrose) at −20°C until use.

### Immunofluorescence

For immunohistochemistry, frozen sections were dried at ambient temperature for 5 min followed by postfixation with −20°C cold methanol for 10 min. Slides were rinsed with PBS and blocked with 5% normal serum (from the species which was used for raising the secondary antibody) for 30 min and incubated with primary antibodies over night at 4°C and secondary antibodies at room temperature for one hour. Antibodies used were: anti-Neurofilament-M (2H3, Developmental Studies Hybridoma Bank), anti-active Caspase-3 (#9664; Cell Signaling), anti-PKA substrate (RRXS/T, #9624; Cell Signaling).

Fixed brain sections were labelled free-floating in primary antibodies overnight at 4°C, secondary antibodies at room temperature for three hours. Antibodies were diluted in PBS containing 0.3% Triton-X. Antibodies used were: anti-Calretinin (6B3, SWANT), anti-doublecortin DCX (sc-8066; Santa Cruz), anti-GFP (ab290; abcam), secondary antibodies: Alexa-555 and Alexa-488 (Invitrogen - Molecular Probes). Nuclei were stained with Sytox or Hoechst stain.

### BrdU labelling

To measure proliferation, mice were injected with BrdU 50 mg/kg i.p. once daily for 3 days as previously described ([49]). The hippocampus was dissected, frozen and double labeling was done on free floating cryosections with antibodies directed against BrdU (Clone BU1/75; Accurate Chemical) and anti-doublecortin (sc-8066; Santa Cruz). Before labelling sections were denatured with 2 M HCl for 10 min and incubated in 0,1 M borate buffer for 10 min. Quantification of labelled cells was done as described ([27]). One-in-seven of 40 µm thick serial sections (280 µm apart) from each brain was immunohistologically stained (see above) and analyzed by fluorescent confocal microscopy. Immunolabelled cells were counted using a 40× objective (Zeiss). Total number of cells was extrapolated by multiplication of the counted numbers of cells with 60 (60 slices per hippocampus).

### Western Blotting

Protein extracts were prepared from 10 hippocampi, using 100 µl buffer (1% NP-40, 50 mM NaCl, 50 mM Hepes) with “Halt” phosphatase inhibitor cocktail (Pierce) and proteaseinhibitors (aprotinin, leupeptin and pepstatin). Tissue was homogenized using an Ultra-Turrax (IKA) for 15 sec. on ice. After centrifugation supernatant was collected, protein concentration was determined (RotiNanoquant, Roth) and 30 µl (about 80 µg total protein) per lane was separated on a SDS gel. Gels were semi-dry blotted onto nitrocellulose (Pall). and were incubated with antibodies against LKB1 (D-19; Santa Cruz), anti-phospho-LKB1 (Ser 431; Santa Cruz) and anti-GAPDH (6C5; Santa Cruz).

### Assay for apoptosis and dying neurons

Apoptosis was detected immunohistochemically by staining for active (cleaved) caspase-3 on frozen sections, Fluoro-Jade-C (Histo-Chem) staining was performed on fresh frozen, unfixed slices as adapted from [50].

### Electron microscopy

Mice were anaesthetized and transcardially perfused according to local institutional guide lines in 3 steps slightly modified according to [51] with 3% paraformaldehyde, 3% glutaraldehyde, 0,5% picric acid in 0.1 M sodium phosphate buffer, pH 7.4 for 10 minute. The hippocampus was dissected and fixed in the same solution for additional 1–2 hours at 4°C, postfixed in 2% osmium tetroxide (2 h, 4°C) and embedded in *Araldite*. For light microscopical identification of target region sections of 1.5 µm thickness were stained with Richardson's blue (1% w/v methylene blue, 1% w/v Azur II) for 1 min, 80°C. 60–80 nm sections (stained for 40 min in uranyl acetate and 7 min in lead citrate) were used for electron microscopy (Zeiss EM 109).

Only those boutons were examined that established contacts with dendritic excrescences of CA3 pyramidal neurons. For quantification of mossy fiber boutons at least 8 independent, non-overlapping analog photographs of the *stratum lucidum* were made at a primary magnification of 3440× of coded sections by an unbiased observer. Each photograph represented an area of 360 µm^2^. Care was taken to avoid areas containing structures not of interest e.g. nuclei or bigger blood vessels. High resolution scans of negatives were made with an EPSON 4990 photo scanner, these scans could be loss-free zoomed in up to 12× and were evaluated with Adobe-Photoshop. To quantify the number of synaptic contacts per section of a bouton, 40 mossy-fiber boutons per animal (n = 3) were evaluated. Synaptic contacts, in contrast to puncta adherentia, were characterized if applicable by 3 criteria: presence of synaptic vesicles close to the presynaptic density, asymmetry between pre- and postsynaptic density and widening of the synaptic cleft. Due to non-ideal orientation of the synaptic cleft, often only the first criterion was applicable.

To determine numbers of synaptic vesicles per bouton, 25 photographs per animal (n = 3) were taken at a primary magnification of 13.000×. Evaluation of eight different areas per animal, each 360 µm^2^ was done. Areas were measured with IMAGE J (NIH; Washington, D.C.) and vesicles were counted with the Image J cell counter plug-in. Statistical evaluation was performed with the Mann-Whitney-U-test.

### Neuronal culture and transfection

Cultures of dissociated hippocampal neurons were prepared and transfected as described previously [52]. Briefly, the hippocampus was dissected from E18 rat embryos, dissociated, and neurons plated onto glass coverslips coated with polyornithine (Sigma) at a density of 800,000 cells per coverslip. Attached neurons were transfected 2 h after plating using Lipofectamine 2000 (Invitrogen) as described. After an incubation for 2 h, the transfection medium was replaced by Neurobasal medium (supplemented with B27, 0.5 mM glutamine, and 100 U/ml penicillin/streptomycin; Invitrogen). The cells were detached after the transfection by moderate pipetting and replated onto new coverslips at a lower density (40,000–60,000 cells per coverslip in a 24-well plate). Neurons were fixed at 3 d.i.v. with 4% paraformaldehyde and 15% sucrose in phosphate buffered saline (PBS) for 20 min at 4°C. To analyse the establishment of neuronal polarity, neurons were stained with the Tau-1 (as a marker for axons) and an anti-MAP2 antibody (minor neurites). Processes showing Tau-1 immunoreactivity in their distal segments were counted as axons, MAP2-positive neurites longer than one cell diameter as dendrites. Three independent transfection experiments were conducted. The following numbers of neurons were analyzed, EGFP: n = 54; p50: n = 37; p65: n = 35; IκB-AA1: n = 42. Means ± s.e.m.; ** p<0.001 compared to EGFP; 3 independent experiments; * p<0.05 compared to EGFP; 3 independent experiments. For the PKAc experiments the following numbers of neurons were analyzed in three independent experiments; EGFP control: n = 46; PKAc: n = 58; IκB-AA1: n = 64; PKAc+IκB-AA1: n = 56.

### Analysis of neuronal morphology

Neuronal morphology was analysed by staining with anti-Tau-1and anti-MAP2 antibodies (Chemicon) and using the WASABI software (Hamamatsu), ImageJ (NIH), and Adobe Photoshop. The development of axonal fate was analyzed as described previously [52]. The length of axons and dendrites was determined by Spot software (Diagnostic Instruments). The Student's *t*-test was used to test statistical significance, when analyzing morphology in vitro.

### Statistical evaluation

For analysis of immunohistological stainings Student's *t*-test was used, Welch's correction was used when indicated in Fig. 6. The evaluation of the behavioral tests occured using two-way ANOVA. Errors bars indicate SEM.; ^*^ *P*<0.05, ^**^ *P*<0.01, ^***^ *P*<0.0001.

### Behavioral assays

#### Spatial pattern separation-Barnes Maze (SPS-BM)

Since behavioral deficits resulting from impairment of neurogenesis may be subtle [27], [53] we designed a new challenging task, the *SPS-BM*. On a circular plate made from hard-plastic (diameter 120 cm) seven rectangular houses of the same colour, size and shape (see rectangles in Fig. 4a) were placed. Only one of the houses was freely accessible and contained a food pellet reward (Kellogs Froot Loops). During seven consecutive days of training (one trial per day, 10 min.) the mice had to find the food house (location F) using distal extramaze cues. Start position is indicated by the letter S. To avoid odour as a facilitatory intramaze cue, the circular plate and the food house was cleaned after each trial with Buraton rapid desinfectant (Schülke & Mayr GmbH). In addition a food pellet is deposited in every house to avoid orientation by odour. Only male mice with an age of six month were tested. Two different genotypes were compared, mice with NF-κB ablation (IκB/tTA, *n* = 9) and control mice (IκB/-, *n* = 8). Habituation occurred one day before starting the task, during 10 minutes all houses were freely accessible and the animals had time to explore the environment and the houses. The performance of the mice was documented by a video-tracking system (TSE VideoMot 2, Bad Homburg) and analyzed by measuring latency, errors and distance covered before arriving at the food house.

#### Classic Barnes Maze test (BM)

The BM was developed to test the ability of spatial learning of rodents [26]. Around the perimeter of a circular plate made out of hard-plastic (diameter 120 cm) there are forty circular holes, one having a tunnel where the mouse has the possibility to hide. During eight consecutive days of training (one trial per day, 30 min) the mice had to find the tunnel with the help of distal extramaze cues. Start position is indicated by the letter S. To avoid odour as a facilitatory intramaze cue, the circular plate and the tunnel was cleaned after each trial with Buraton. Only six months old males were tested. Two different genotypes were compared, mice with NF-κB ablation (IκB/tTA, *n* = 7) and control mice (IκB/-, *n* = 7). Habituation took place one day before starting the test. During that phase animals had ten minutes to explore the environment without the tunnel. Performance was documented and evaluated as defined above.

## Supporting Information

Figure S1 **Mouse Model.** Two different transgenic lines (CamKII/tTA and tetO/IκB-AA1-GFP) were crossbred and double transgenic animals (IκB/tTA) were analysed in comparison to controls. Doxycycline (Dox) treatment inhibits the expression of IκB and GFP.
(TIF)

Figure S2 **Transgene expression of IκB and GFP starts in type 2b immature neurons.** Distribution of doublecortin (DCX) and GFP in the hippocampus. Colocalisation of DCX and GFP shows transgene expression within type 2b neuronal granule cell precursors. Note that control mice (IκB/-) do not express the transgenic GFP. bar 20 µm; DG – granular layer, ML – molecular layer
(TIF)

Figure S3 **Ultrastructure of mossy fiber boutons in (a) controls (IκB/-) and (b, c) IκB/tTA mice.** (**a**) A mossy-fiber bouton (mfb) of a control mouse filled with synaptic vesicles indented by 3 spiny excrescences (se), the shaft of the dendrite (de) is contacted by several puncta adherentia (arrowheads). Inset: Three synaptic contacts (white asterisks) on one of the excrescences. black asterisks mitochondria. (**b**, **c**) IκB/tTA mice (**b**) Terminal region of a mossy fiber (mf) ending in a bouton with one spiny excrescence (se) visible. (**c**) Mossy fiber bouton contacted by 2 spiny excrescences (se). Note significant smaller size of boutons in super-repressor mice. black asterisks mitochondrium, white asterisks synaptic contact. All photographs to same scale; Bar: 0.5 µm, inset bar: 0.25 µm (**d**) Number of boutons (mossy fiber boutons: MFB) were significantly reduced (38% reduction) in IκB/tTA mice. (**e**) Numbers of synaptic contacts per mossy fiber bouton were also significantly reduced by ca. 30%. (**f**) No difference in the number of synaptic vesicles per unit of area (µm^2^) could be observed. Error bar: SEM; (*** p≤0.001).
(TIF)

Figure S4 **PKA expression results in the formation of supernumerary axons.** (**b**) Expression of the super-repressor-IκB reduced the number of axons significantly (0.5 axons per cell±0.3) as compared to control transfections (**a**). In contrast, overexpression of PKAc in hippocampal neurons resulted in hyperpolarized neurons with multiple axons (**c**) consistent with the recent observation of PKA dependent phosphorylation of the downstream kinase LKB1, which is necessary for axon differentiation. Coexpression of super-repressor-IκB and PKAc led to a hyperpolarized phenotype (**d**). Successful transfected cells were identified via EGFP fluorescence.
(TIF)

Figure S5 **Comparison between the Classic Barnes Maze (BM) and the spatial pattern separation Barnes Maze (SPS-BM) and the underlying neuronal circuits. Spatial pattern separation Barnes Maze (SPS-BM).** (**A**) We designed a new challenging task for measuring impairments in neurogenesis, the *SPS-BM*. On a circular plate seven rectangular houses of the same colour, size and shape were placed. Only one of the houses was freely accessible and contained a food pellet reward. During seven consecutive days of training (one trial per day, 10 min.) the mice had to find the food house (location F) using distal extramaze cues. Start position is indicated by the letter S (for details see Material and Methods). (**B**) The SPS-BM is a dentate gyrus dependent task which relies on a functional trisynaptic circuit from ECII – GD-CA3 -CA1 and EC VI, thus changes in neurogenesis, mossy fiber pathway or dentate gyrus tissue homeostasis can be easily measured (see Fig. 5 in the main manuscript). (**C**) Classic *Barnes Maze (BM)* Around the perimeter of a circular plate one of forty holes is attached to a tunnel (arrow) in which the mouse can hide. During eight consecutive days of training the mouse has to find this tunnel with the help of distal extramaze cues (S = Start position), for details see Materials and Methods. (**D**) The classic Barnes Maze is a dentate gyrus independent task which mainly relies on navigation via the monosynaptic temporoammonic pathway from ECIII-CA1-EC V. (EC = enthorinal cortex layers).
(TIF)

Figure S6 **A Classic Barnes Maze (BM) was used to test the principal learning ability of the transgenic lines.** Two genotypes were compared, mice with NF-κB ablation (IκB/tTA, *n* = 7) and control mice (IκB/-, *n* = 7). Their performance was evaluated by measuring latency (**a**), errors (**c**) and distance covered (**b**) until the tunnel was reached. In all parameters monitored in the classic Barnes Maze test, no significant difference between the two groups was observed.
(TIF)

Figure S7 **Search strategy in the spatial pattern separation Barnes Maze (SPS-BM).** (**a**, **d**) In control and NF-κB ablated groups a random search strategy is used in the beginning of the task. (**b**, **e**) After several days of testing control mice preferred a serial search strategy, whereas IκB/tTA mice use random and serial search strategies by chance. (**c**, **f**) At the end of the task, only control animals were able to use the spatial strategy and move straight forward to the food house, indicating the successful learning process. **a–f**: representative examples of computerized trackings.
(TIF)
