# Supplementary figures and images for: Regrowing the Adult Brain: NF-κB Controls Functional Circuit Formation and Tissue Homeostasis in the Dentate Gyrus
Source: PLoS One. 2012 Feb 1;7(2):e30838. doi: 10.1371/journal.pone.0030838 (PMC3270021; doi:10.1371/journal.pone.0030838)

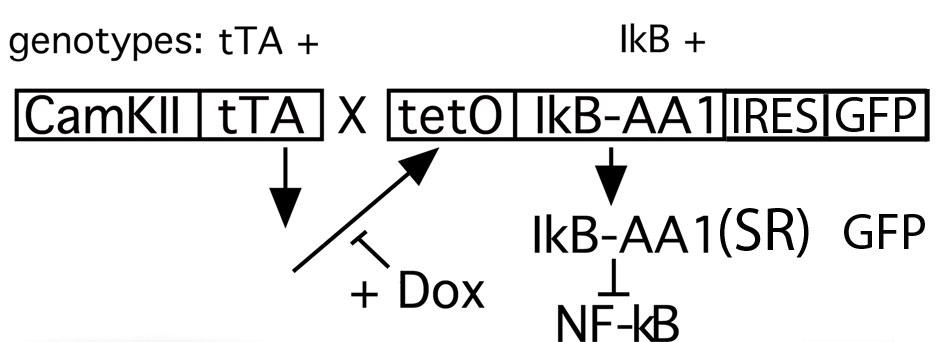

Supplement: Figure S1 — Mouse Model. Two different transgenic lines (CamKII/tTA and tetO/IκB-AA1-GFP) were crossbred and double transgenic animals (IκB/tTA) were analysed in comparison to controls. Doxycycline (Dox) treatment inhibits the expression of IκB and GFP. (TIF) [file pone.0030838.s001.tif]

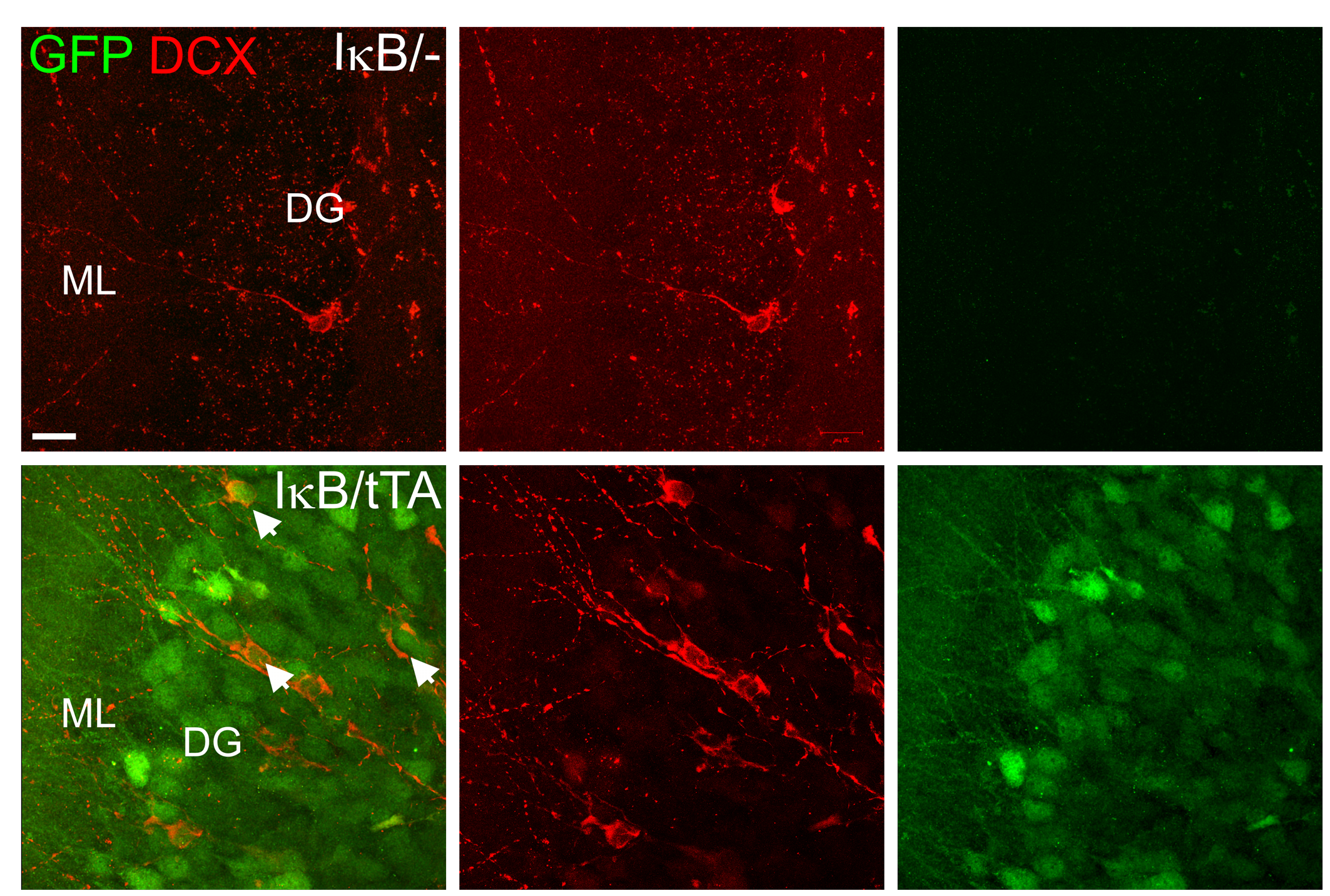

Supplement: Figure S2 — Transgene expression of IκB and GFP starts in type 2b immature neurons. Distribution of doublecortin (DCX) and GFP in the hippocampus. Colocalisation of DCX and GFP shows transgene expression within type 2b neuronal granule cell precursors. Note that control mice (IκB/-) do not express the transgenic GFP. bar 20 µm; DG – granular layer, ML – molecular layer (TIF) [file pone.0030838.s002.tif]

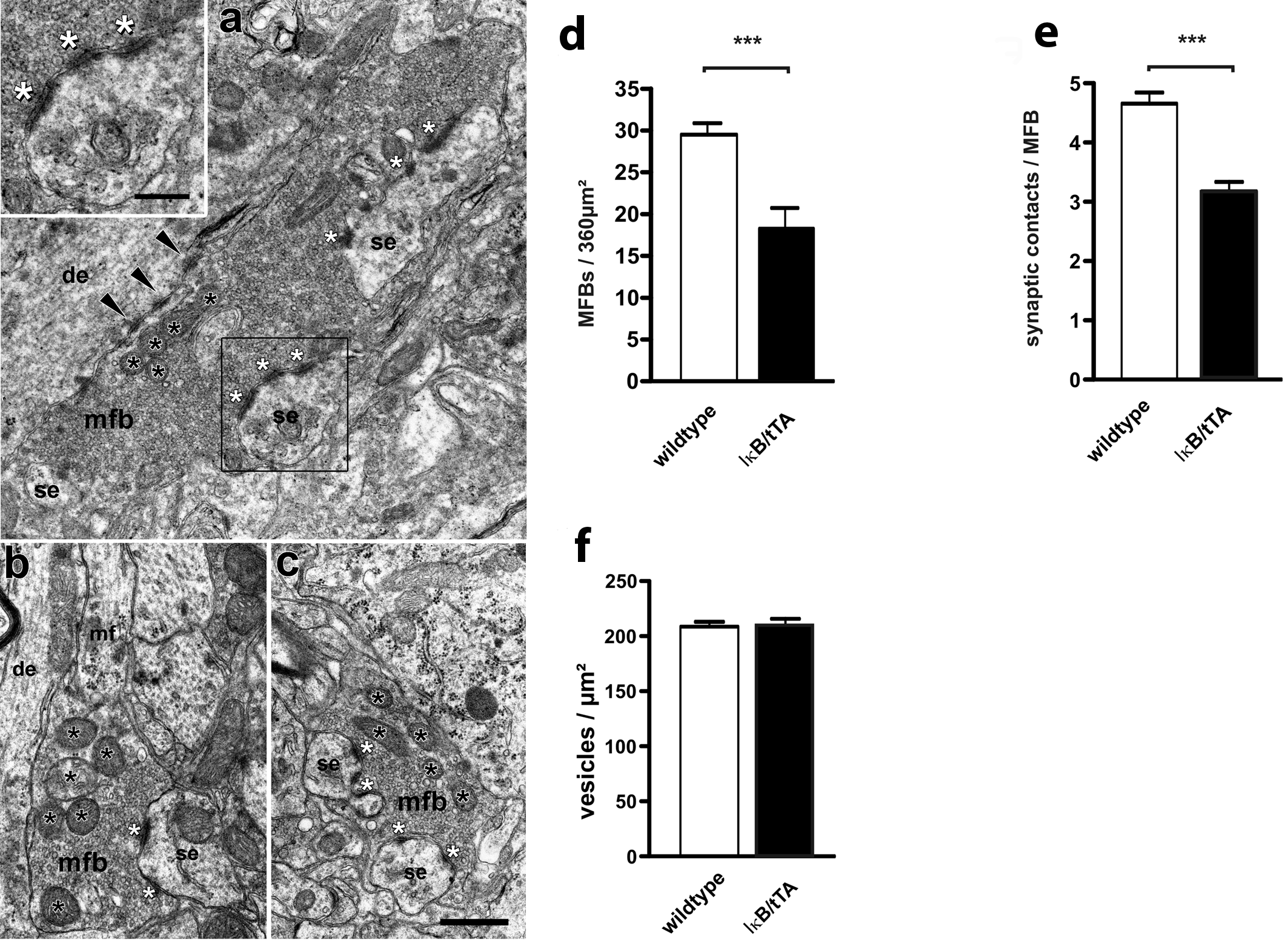

Supplement: Figure S3 — Ultrastructure of mossy fiber boutons in (a) controls (IκB/-) and (b, c) IκB/tTA mice. (a) A mossy-fiber bouton (mfb) of a control mouse filled with synaptic vesicles indented by 3 spiny excrescences (se), the shaft of the dendrite (de) is contacted by several puncta adherentia (arrowheads). Inset: Three synaptic contacts (white asterisks) on one of the excrescences. black asterisks mitochondria. (b, c) IκB/tTA mice (b) Terminal region of a mossy fiber (mf) ending in a bouton with one spiny excrescence (se) visible. (c) Mossy fiber bouton contacted by 2 spiny excrescences (se). Note significant smaller size of boutons in super-repressor mice. black asterisks mitochondrium, white asterisks synaptic contact. All photographs to same scale; Bar: 0.5 µm, inset bar: 0.25 µm (d) Number of boutons (mossy fiber boutons: MFB) were significantly reduced (38% reduction) in IκB/tTA mice. (e) Numbers of synaptic contacts per mossy fiber bouton were also significantly reduced by ca. 30%. (f) No difference in the number of synaptic vesicles per unit of area (µm2) could be observed. Error bar: SEM; (*** p≤0.001). (TIF) [file pone.0030838.s003.tif]

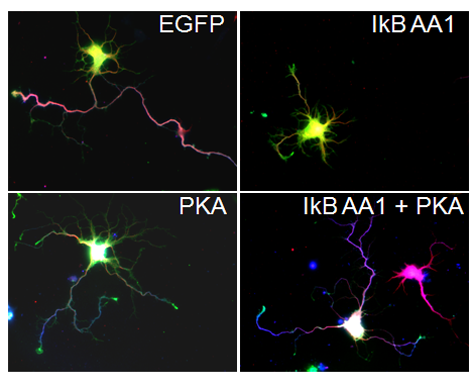

Supplement: Figure S4 — PKA expression results in the formation of supernumerary axons. (b) Expression of the super-repressor-IκB reduced the number of axons significantly (0.5 axons per cell±0.3) as compared to control transfections (a). In contrast, overexpression of PKAc in hippocampal neurons resulted in hyperpolarized neurons with multiple axons (c) consistent with the recent observation of PKA dependent phosphorylation of the downstream kinase LKB1, which is necessary for axon differentiation. Coexpression of super-repressor-IκB and PKAc led to a hyperpolarized phenotype (d). Successful transfected cells were identified via EGFP fluorescence. (TIF) [file pone.0030838.s004.tif]

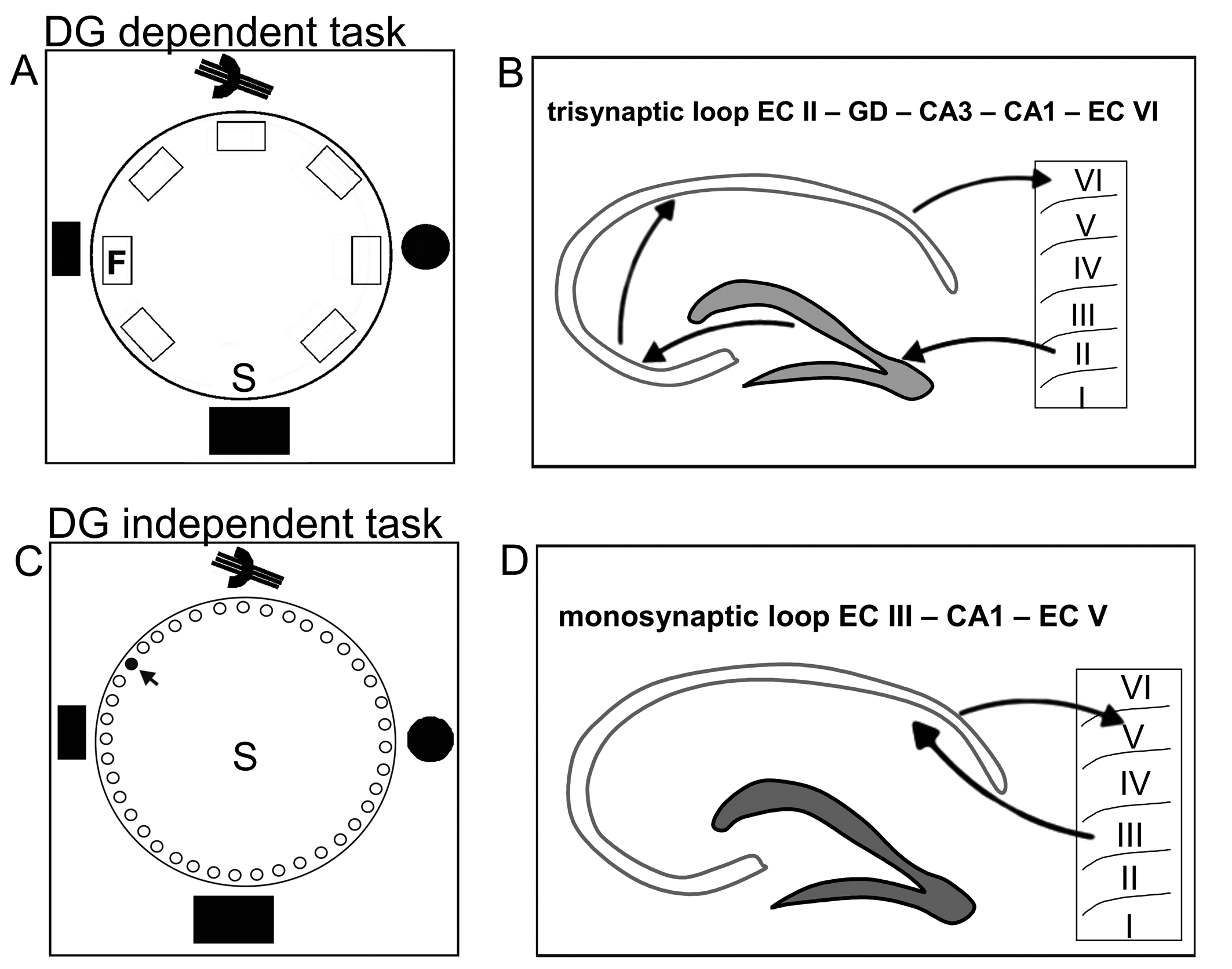

Supplement: Figure S5 — Comparison between the Classic Barnes Maze (BM) and the spatial pattern separation Barnes Maze (SPS-BM) and the underlying neuronal circuits. Spatial pattern separation Barnes Maze (SPS-BM). (A) We designed a new challenging task for measuring impairments in neurogenesis, the SPS-BM. On a circular plate seven rectangular houses of the same colour, size and shape were placed. Only one of the houses was freely accessible and contained a food pellet reward. During seven consecutive days of training (one trial per day, 10 min.) the mice had to find the food house (location F) using distal extramaze cues. Start position is indicated by the letter S (for details see Material and Methods). (B) The SPS-BM is a dentate gyrus dependent task which relies on a functional trisynaptic circuit from ECII – GD-CA3 -CA1 and EC VI, thus changes in neurogenesis, mossy fiber pathway or dentate gyrus tissue homeostasis can be easily measured (see Fig. 5 in the main manuscript). (C) Classic Barnes Maze (BM) Around the perimeter of a circular plate one of forty holes is attached to a tunnel (arrow) in which the mouse can hide. During eight consecutive days of training the mouse has to find this tunnel with the help of distal extramaze cues (S = Start position), for details see Materials and Methods. (D) The classic Barnes Maze is a dentate gyrus independent task which mainly relies on navigation via the monosynaptic temporoammonic pathway from ECIII-CA1-EC V. (EC = enthorinal cortex layers). (TIF) [file pone.0030838.s005.tif]

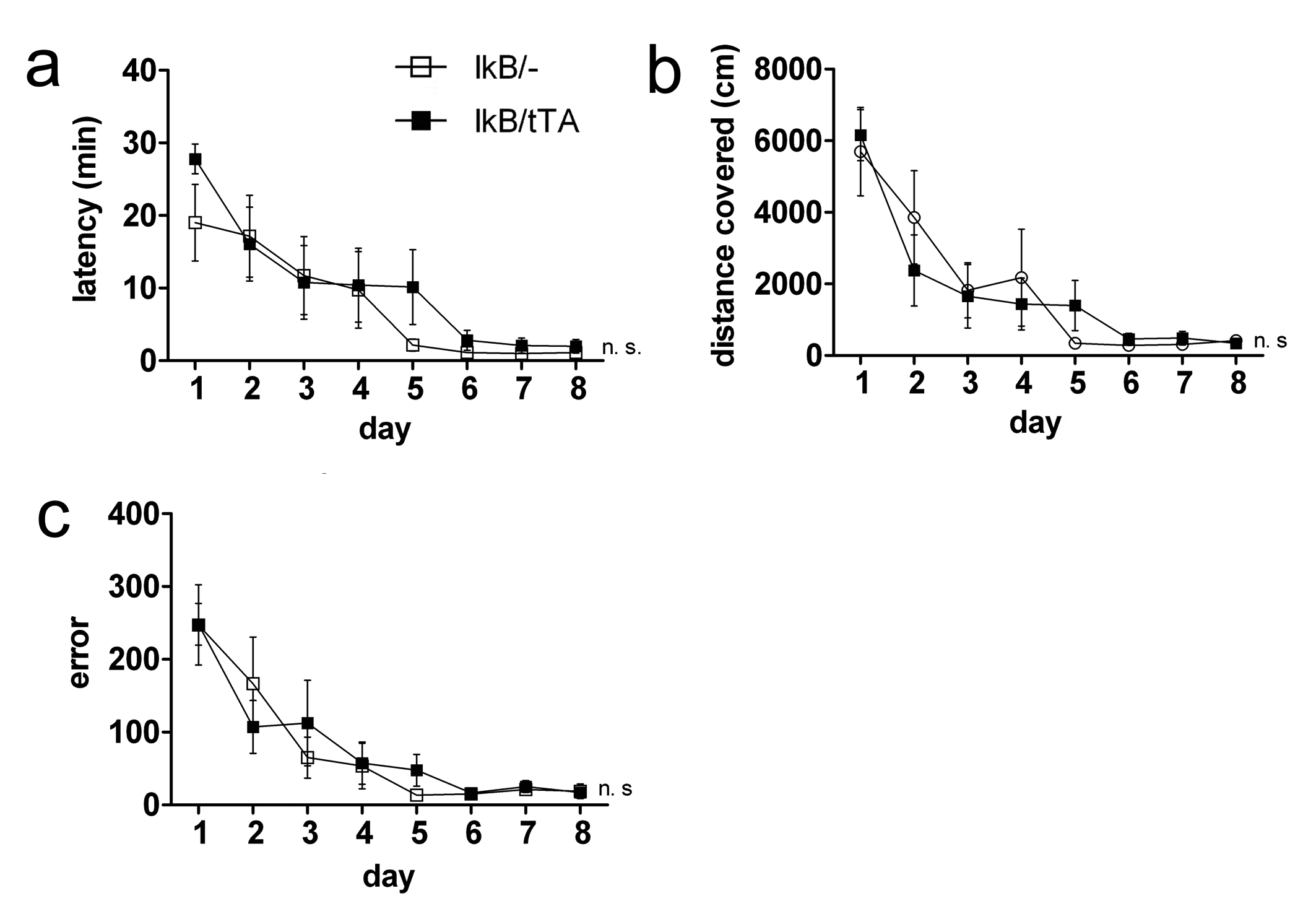

Supplement: Figure S6 — A Classic Barnes Maze (BM) was used to test the principal learning ability of the transgenic lines. Two genotypes were compared, mice with NF-κB ablation (IκB/tTA, n = 7) and control mice (IκB/-, n = 7). Their performance was evaluated by measuring latency (a), errors (c) and distance covered (b) until the tunnel was reached. In all parameters monitored in the classic Barnes Maze test, no significant difference between the two groups was observed. (TIF) [file pone.0030838.s006.tif]

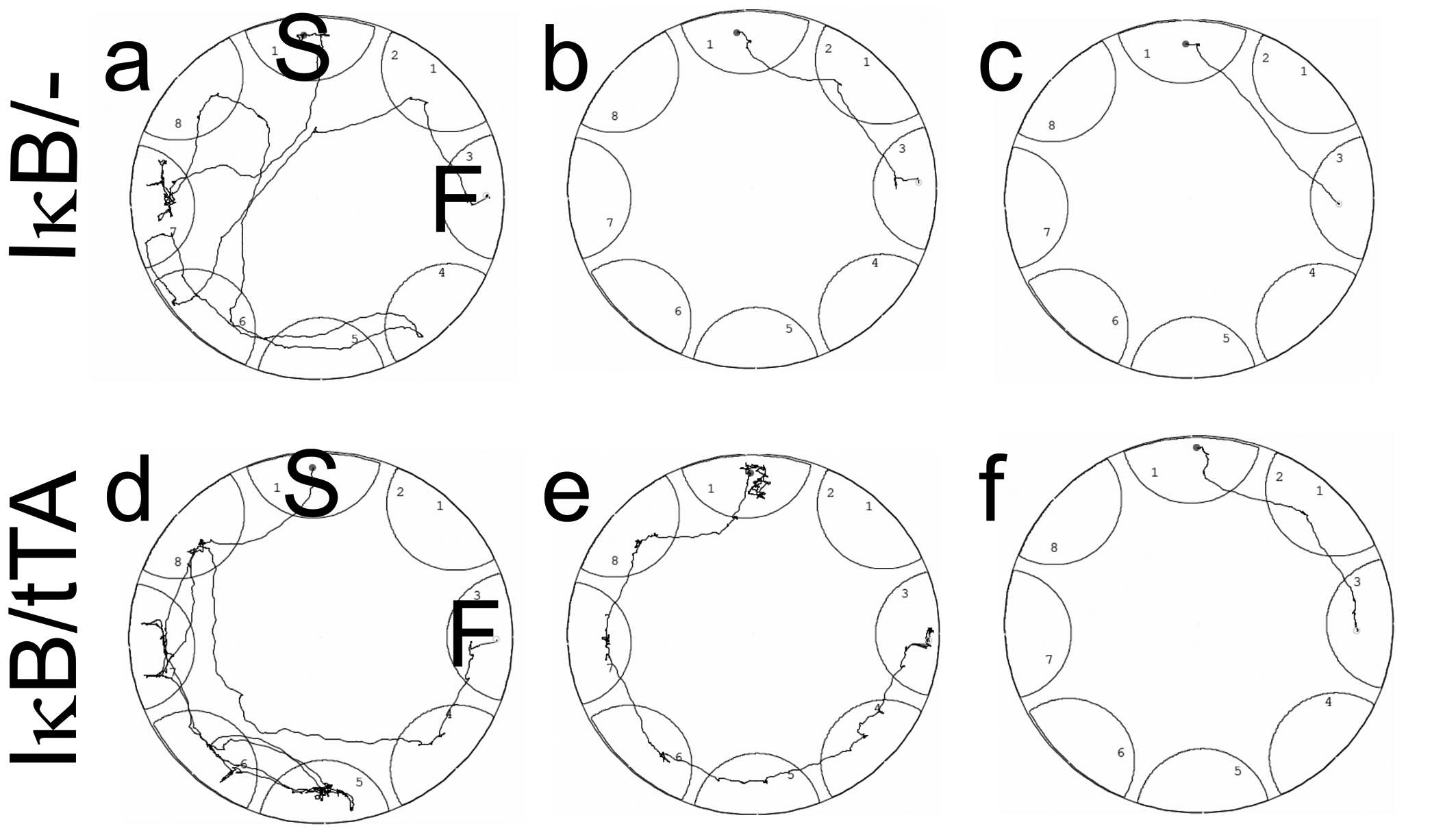

Supplement: Figure S7 — Search strategy in the spatial pattern separation Barnes Maze (SPS-BM). (a, d) In control and NF-κB ablated groups a random search strategy is used in the beginning of the task. (b, e) After several days of testing control mice preferred a serial search strategy, whereas IκB/tTA mice use random and serial search strategies by chance. (c, f) At the end of the task, only control animals were able to use the spatial strategy and move straight forward to the food house, indicating the successful learning process. a–f: representative examples of computerized trackings. (TIF) [file pone.0030838.s007.tif]
